# Metallic Nanoparticle-Based Optical Cell Chip for Nondestructive Monitoring of Intra/Extracellular Signals

**DOI:** 10.3390/pharmaceutics12010050

**Published:** 2020-01-07

**Authors:** Sang-Nam Lee, Jin-Ha Choi, Hyeon-Yeol Cho, Jeong-Woo Choi

**Affiliations:** 1 Department of Chemical & Biomolecular Engineering, Sogang University, 35 Baekbeom-ro, Mapo-Gu, Seoul 04107, Korea; snlee9191@hanmail.net (S.-N.L.); jinhachoi@sogang.ac.kr (J.-H.C.); 2 Uniance Gene Inc., 1107 Teilhard Hall, 35 Baekbeom-Ro, Mapo-Gu, Seoul 04107, Korea

**Keywords:** cell chip, metallic nanoparticles, biosensors, intracellular signal, extracellular signal, nondestructive monitoring

## Abstract

The biosensing platform is noteworthy for high sensitivity and precise detection of target analytes, which are related to the status of cells or specific diseases. The modification of the transducers with metallic nanoparticles (MNPs) has attracted attention owing to excellent features such as improved sensitivity and selectivity. Moreover, the incorporation of MNPs into biosensing systems may increase the speed and the capability of the biosensors. In this review, we introduce the current progress of the developed cell-based biosensors, cell chip, based on the unique physiochemical features of MNPs. Mainly, we focus on optical intra/extracellular biosensing methods, including fluorescence, localized surface plasmon resonance (LSPR), and surface-enhanced Raman spectroscopy (SERS) based on the coupling of MNPs. We believe that the topics discussed here are useful and able to provide a guideline in the development of new MNP-based cell chip platforms for pharmaceutical applications such as drug screening and toxicological tests in the near future.

## 1. Introduction

Recent advances in biotechnology and pharmaceutics have revealed new possibilities for the development of drugs with improved performance based on precise and sensitive analyses [1,2,3,4,5,6]. To evaluate the working efficiency of drug candidates on a target disease, the development of in vitro nondestructive cellular signal monitoring techniques is required. To this end, the cell chip platform has been developed, which consists of a transducer that converts cellular signals to optical signals [7,8,9]. Nanomaterials have been widely utilized for the fabrication of biosensors and biochips due to their prominent features, such as high conductivity, large surface area, and excellent mechanical and optical features [10,11,12,13]. They are considered one of the most attractive materials to develop new methods in the construction of next-generation biosensors and sensors. Different kinds of nanomaterials, including carbon nanotubes, gold nanoparticles, magnetic nanoparticles, and graphene, have been used in biosensor construction for pharmaceutical analysis [14,15,16,17]. Notably, the sensitivity of biosensors has improved tremendously because of the incorporation of nanomaterials in biosensor fabrication.

Among these nanomaterials, metallic nanoparticles (MNPs) have garnered significant interests for intra/extra cellular signal detection using their unique physical and chemical properties [18,19,20]. Particularly, MNPs can enhance the signal intensity of the target biomolecules from the cells using their specific properties related to the high density of electrons on the MNPs [21,22,23]. Furthermore, the ease of surface modification with a wide range of biomolecules, such as oligonucleotides and proteins, enables the development of novel sensing and chip platform with improved performances in the detection of various biomolecules from the cells [24,25,26]. One of the most significant properties of the MNPs for biosensor applications is their electromagnetic (EM) fields generated by free electrons around nanoparticles. This property can induce localized surface plasmon resonance (LSPR), which is induced by the irradiation of light and the excited free electrons simultaneously producing collective consistent oscillations [27,28,29,30,31]. LSPR on MNPs such as Au and Ag exhibited unique features underlying their strong absorption and scattering of light, making MNPs attractive candidates for the nanoprobes of the several biomolecules. Moreover, the EM field around MNPs results in the amplification of surface-enhanced Raman scattering (SERS) [32,33,34,35]. In addition, fluorescent signals can also be quenched or enhanced due to the energy transfer between the MNPs and fluorophore [36,37,38]. Moreover, MNP’s plasmonic effects can be tuned by selecting the optimal wavelength of the light source and exposure time to minimize a photothermal effect which can give a damage on the bio-analytes [39,40]. These great properties have led to the development of biosensors for in vitro cellular analysis on a cellular level and diagnosis of specific diseases at the molecular level.

In this review, the optical properties of novel MNPs and their application on the cell chip platforms for intra/extra cellular signal detection will be discussed. Various types of MNPs exhibit interesting surface and interface features, which significantly improves the biocompatibility and transduction of the biosensor in comparison to the same process in the absence of these MNPs. Regarding this, each section individually focuses on one of the following biosensors: Fluorescence, LSPR, and surface-enhanced Raman spectroscopy (SERS) based on the coupling of MNPs and cellular components.

## 2. Fluorescence-Based Analytical Platform on Cell Chip Using MNPs

Among biomolecular analytical methods, the fluorescence method is an excellent and widely used technique for the recognition of biological status and changes at the intercellular and intracellular levels [41,42,43,44]. For example, cell surface markers have been characterized by specific primary antibodies and fluorescent dye-labeled secondary antibodies [45]. These fluorescence images have provided cell-specific information. The advantages of fluorescence-based analytical methods include high sensitivity, selectivity, and reproducibility [46,47,48,49]. Furthermore, fluorescence detection can be easily applied to the inside and outside of cells for noninvasive and multi-analytes detection [50,51,52]. Based on these features, fluorescence methods can be integrated into the cell chip platform to enable research on in vitro drug screening or toxicological tests related to diseases, stem cell differentiation, and apoptosis on the cell level (Table 1). The fluorescence-based biomolecular analytical system combined with cell chip platforms makes it possible to develop high-throughput screening and analytical systems. In addition, the fluorescence-based system has been pointedly improved by metallic nanomaterials, which provide recognition of the interaction between a target on the cell and signal transduction from biomolecular events (e.g., binding of antigen-antibody) to fluorescent signals. MNPs can be easily functionalized for biomolecular detection processes using their specific properties such as quenching and metal-enhanced fluorescence effects, making them excellent platforms for sensing at the cell level [39,53,54,55,56].

### 2.1. Metallic Nanoparticle-Based Fluorescence Sensing Strategies for Intracellular Signal Detection

Recently, many researchers have sought to develop an in situ fluorescence analysis strategy for sensing diverse biological samples such as DNA, RNA, and proteins using MNPs [57,58,59,60,61]. Representatively, Au nanoparticles have frequently been used for biomolecular detection by the fluorescence resonance energy transfer (FRET) effect [62]. In FRET, the electronic excitation energy of a fluorescent dye (donor) is transferred to a MNP (acceptor), and then the donor cannot emit light. If the MNP is composed of noble elements such as Au and Ag, the emitted light is not generated, which we call the quenching effect. On the other hand, emission light can be generated if the nanoparticle acceptors have fluorescent properties such as quantum dot (QD) and upconversion nanoparticle (UCNP) [63,64]. For activation of this phenomenon, there is a specific condition between fluorescent donor and acceptor. MNPs should be presented close (below a few nanometers) to the fluorescent donor for the energy transfer. Therefore, the change of the distance could be caused by the alteration of both (donor and acceptor) emission intensities. The biomolecules could induce the change of distance through binding or dissociating events such as DNA hybridization or enzymatic cleavage reaction. Therefore, the FRET-based analytical method has a high potential for biomolecular detection due to its sensitivity and simplicity. Liu et al. exhibited a FRET-based sensor for the detection of multi-microRNAs (miRNAs) for the early diagnosis of lung cancer using fluorophore–quencher pairs [65]. However, the traditional fluorophore–quencher pairs are exposed to analytes, the separation between the donor and quencher provides the fluorescence signals, and the single fluorescent signal can only provide low sensitivity in detection [66]. Therefore, the MNPs, which could be great donors and quenchers, have been used in FRET probes for improving detection sensitivity and increasing sensing range.

**Table 1:** Fluorescence-based analytical platforms for cell analysis with metallic nanomaterials.

Metallic Nanomaterials		Mechanism	Target	Function	Ref.
**Solution-based metallic nanoparticles**	Au nanoparticle, Quantum dot	Fluorescence resonance energy transfer (FRET), Dequenching-Quenching	miR-21	Differentiation between cancer cells and normal cells miRNA detection with spatiotemporal control in living cells	[65,66]
	Au nanocross, Au nanorod	Fluorescence resonance energy transfer and surface-enhanced fluorescence (FRET-SEF)	miR-34a	Measurement of miRNA-34a from the HepG2 and H9C2 cells stimulated by AFB1 and TGF-β1	[67]
	Au nanoparticle	FRET, hairpin-locked-DNAzyme system	miR-141	Amplified detection of miR-141 by 25pM in living cells	[68]
	Au nanoparticle	FRET, aptazyme	Adenosine triphosphate (ATP)	Entering living cells and recognize intracellular target detection	[69]
**Nano-platform onto the cell chip**	Nanostructured plasmonic gold (pGold) chip (Au nanorods)	Fluorescence enhancement on a plasmonic substrate	Tubulin, HER2, EGFR	Multiple cellular proteins of single cells of various cell types can be detected through a microarray of cells	[70]
	Au nanoarray chip (Nano-sized holes)	Fluorescence enhancement on a plasmonic substrate	EpCAM	Fluorescence enhancement of APC-EpCAM in the cell membrane in contact with the plasmonic chip	[71]

**Figure 1:**
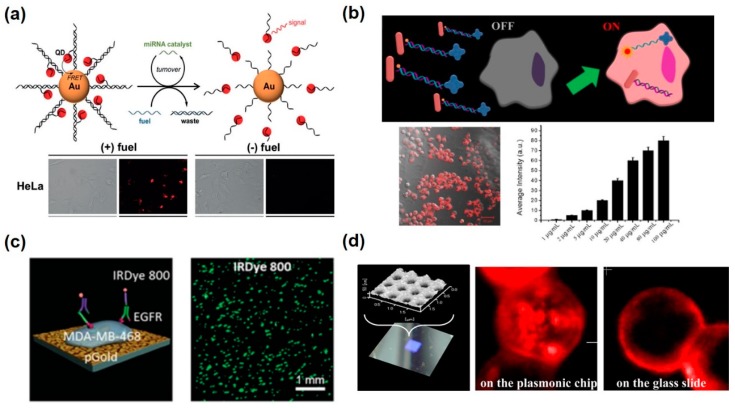
Fluorescence-based intracellular and extracellular analysis using metallic nanomaterials. (**a**) Au nanoparticle-quantum dots (QDs) nanoassembly for catalytic intracellular miRNA sensing with fuel DNA strands. Reproduced with permission [67]. Copyright 2015, ACS Publications. (**b**) “OFF-Enhanced ON” fluorescent switching system for the specific detection of miRNAs in intact cancer cells. Reproduced with permission [69]. Copyright 2018, ACS Publications. (**c**) Plasmonic gold chip with nanostructures for the metal enhanced fluorescence-induced immunofluorescence detection. Reproduced with permission [72]. Copyright 2016, WILEY-VCH. (**d**) Au plasmonic chip consisted of two-dimensional periodic structure for the enhanced fluorescence effect. Reproduced with permission [73]. Copyright 2016, ACS Publications.

The solution-based FRET sensing systems have mainly been studied for the detection of biological changes such as miRNA expression levels inside the cells. He et al. developed a DNA-functionalized Au nanoparticle-QD complex for the detection of miRNAs related to cancer in living cells through FRET-based fluorescence measurement [67] (Figure 1a). They claimed that the Au-QD nanoparticles could provide catalytic imaging of cancer-related miRNAs in living cancer cells with high sensitivity. The miR-21 induced catalytic disassemble reaction of QD from the Au nanoparticle using the thermodynamically driven process, and it enabled improved sensitivity by three orders of magnitude, comparing non-catalytic Au nanoparticle-QD complex. Another FRET-based miRNA sensor was studied using photocaged nanoparticle in the same research group [68]. After the Au nanoparticle-QD complex was internalized to the cancer cell for the detection of miRNA, the complex was activated by the degradation of a photocleavable group using UV irradiation. Thus, it could detect miRNA in the living cell with temporal control. Sun et al. successfully conducted miR-34s detection in HepG2 and H9C2 cells with two different Au nanoparticles and one fluorophore by FRET effect [69] (Figure 1b). Au nanorod and Au nanocross were connected by the fluorophore-tagged double-stranded DNA at the surface of the Au nanorod. In this case, the fluorophore should be quenched by the Au nanorod. Targeted miRNA (miR-34a) could be attached to the complementary DNA on the Au nanorod, and fluorophore-tagged DNA was detached from the Au nanorod. Therefore, fluorescence intensity should be increased, whereby miR-34a could be measured by the fluorescence emission. Moreover, fluorescence was enhanced by the metal enhanced fluorescence effect from the Au nanocross. The authors claimed that it was an ultrasensitive miRNA sensing system with a detection limit of 0.5 aM and 0.03 fM. The developed nanoprobe could monitor the endogenous miRNA expression in living cells dynamically. Similarly, Yang et al. developed an aptazyme-based ATP sensor using Au nanoparticles. An aptazyme is a DNA complex that consists of a DNA aptamer and a DNAzyme [70]. In this system, the DNAzyme can only be activated by the target binding to the DNA aptamer. Using this recognition-activation process, aptazymes could be applied to the miRNA sensing system with high selectivity and sensitivity by the multiple-turnover reactions for signal amplification. In addition, the same research group designed an Au nanoparticle-based DNAzyme nanoprobe for miRNA detection in living cells [71]. A hairpin-DNAzyme complex was functionalized on the Au nanoparticle, and the fluorophore, which was positioned to the end of the hairpin, was quenched. Once the target miRNA was bound to the hairpin DNA, its structure was changed to the active secondary structure whose catalytic core cleaved the self-strand DNA. Therefore, miRNA could be quantified by the measurement of fluorescence intensity. In addition, the target miRNA could release and bind to another hairpin-DNAzyme complex to induce another cycle of activation. This amplification strategy provided signal enhancement and highly sensitive detection, ranging from 200 pM to 150 nm, of miR-141. In addition to the Au nanoparticle, other nanomaterials have been used for neurotransmitter detection. UCNPs played the role of fluorescence emission, and graphene oxide exhibited the quenching effect in this system [64]. These particles are connected to each other by the π–π interaction of dopamine aptamer, and the dopamine could induce the dissociation of graphene oxide from the aptamer by the aptamer–dopamine binding event. Separated graphene oxide from the upconversion nanoparticle could not hinder the fluorescence emission; therefore, dopamine was determined by the fluorescence signal intensity. Using this system, neuronal differentiation could be monitored by measuring expression of dopamine into the cell through the increase in fluorescence signal.

### 2.2. Fluorescence Detection Platform with Metallic Nanostructures on Cell Chip for Extacellular Signal Detection

The metallic nanostructures integrated fluorescence-based assay could be applied to the cell analysis through cell chip technology. Koh et al. demonstrated that nanostructured Au immobilized on the surface of the chip could induce the fluorescence-enhancing effect due to the surface plasmon resonances and local electrical field enhancement [72] (Figure 1c). This chip could enhance the fluorescence intensity in the near-infrared region, ranging from 500 to 900 nm wavelength. When IRDye 800 was labeled on the Au chip, fluorescence enhancement was found to be the strongest in the 700–900 nm range, increased by up to 33-fold when compared to the dye itself. In addition, multiple surface protein markers of cells could be detected with microarray spotting technology on Au chips. Different protein surface markers (tubulin, HER2, and EGFR) on the cells confirmed the fluorescence enhancement with nuclei staining. This showed the potential of multiplexed single-cell analysis using fluorescence-enhancing effect by metallic plasmonic nanosubstrates. Tawa et al. developed an Au-coated periodic nanoarray for the enhancement of the fluorescence emission of a fluorophore [73] (Figure 1d). Two different cancer cell lines (MDA-MB-231 and MCF-7) were observed on an Au nanoarray under a multicolor fluorescence microscope. In living cells, the fluorescence of EpCAM marked with APC-labeled antibody was ten times brighter on the Au nanoarray, compared to on the glass substrate. These metal nanostructure-based fluorescence chips are expected to be a powerful tool for the analysis of biomolecules at the single cell level.

## 3. LSPR-Based Analytical Platform on Cell Chip Using MNPs

Metallic nanostructures are effective materials for inducing EM fields at the nanoscale [74]. They can induce the LSPR effect by supporting the enhancement of the electron oscillations on the surface of metal nanostructures. The EM field of incident light can easily penetrate the metallic nanostructures and hold the conduction band electrons, resulting in their clear shift away from the positively charged metallic ions. Afterward, the electric dipole pulls the polarized electrons back to the lattice. Therefore, the consistent oscillation of the electrons of the metallic nanostructures can be considered as a choral oscillator driven by the energy resonance of light. Metallic nanostructures, mainly Au and Ag, have been generally applied to the LSPR-inducing nanomaterials for biomolecular analysis in cells due to several advantages [75,76,77]. LSPR-based analytical systems make it possible to obtain crucial information for multiple cellular structures or to interact with biological occurrences simultaneously. In addition, they facilitate visualization of biological processes at the molecular and cellular levels in a noninvasive manner. Compared to the fluorescence-based methods, LSPR-based analytical systems can overcome limitations such as blinking and photobleaching owing to using the inherent optical properties. This section gives an overview of the LSPR-based analytical methods at the cellular level using the metallic nanostructures, besides FRET, MEF, and SERS-based analytical methods, which are closely related to the LSPR effect on the surface of metallic nanostructures (Table 2).

### 3.1. Metallic Nanoparticle-Based LSPR Sensing Strategies onto the Cell for Intracellular Signal Detection

**Figure 2:**
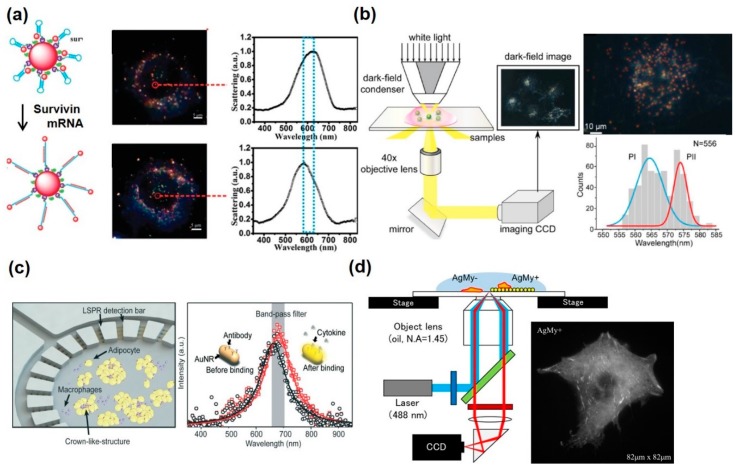
Localized surface plasmon resonance (LSPR)-based intracellular and extracellular analysis using metallic nanomaterials. (**a**) Survivin mRNA analysis using nanoprobes, composed of molecular beacon, small and large Au nanoparticle. Reproduced with permission [85]. Copyright 2016, Royal Society of Chemistry. (**b**) The high-throughput method for studying the resonance scattering light of single plasmonic nanoparticles in a dark-field image for intracellular analysis. Reproduced with permission [86]. Copyright 2015, Ivyspring International Publisher. (**c**) Adipose-tissue-on-chip sensing platform for an in situ multiplexed analysis of adipose tissue inflammation by changing LSPR property of the Au nanorod. Reproduced with permission [87]. Copyright 2018, Royal Society of Chemistry. (**d**) Cell analysis by total internal reflection fluorescence (TIRF) microscope system on Au nanosheets. Reproduced with permission [88]. Copyright 2017, PLOS.

MNPs can emit scattered light due to the resonant interaction between the incident light and the collective oscillation of free conduction electrons. This optical phenomenon by LSPR has attracted significant attention for applications in intracellular sensing and imaging [78,79,80]. Traditional LSPR-based analytical methods are generally based on the aggregation of Au nanoparticles, which results in the color change of the solution. Using this property, lots of colorimetric sensors have been developed to detect biomolecules and heavy metal ions [81,82,83]. There have been intracellular analysis using the Au aggregation effect. Jun et al. developed a core-satellite nanoparticle to measure the caspase-3 level in living cells [84]. Au nanoparticles were connected to each other by the peptide sequence, which could be cleaved by the caspase-3. A color change of the Au nanoparticle solution and the absorbance peaks shift could be observed by the caspase-3 in living cells. Qian et al. demonstrated a survivin mRNA detection using the plasmonic nanohalo complex, consisting of large and small Au nanoparticles [85] (Figure 2a). Two nanoparticles were connected by the molecular beacon, and it could capture the surviving mRNA. The molecular beacon could be straightened, and its optical property thereby changed. This could be easily observed by the color change by naked eye, as well as the red shift of the absorbance peak. In addition, it could be detected by dark-field imaging, which has high signal-to-noise ratio and has been broadly applied in the MNP-mediated LSPR-based detections, with different concentrations of the mRNA, ranging from 10 to 1000 pM. Compared to conventional nanoprobes, dark-field imaging showed higher signal-to-noise ratios with a strong multiple plasmon coupling effect for the detection of biomolecules in situ. Gu et al. showed the high-resolution images of single nanoparticles in the living cells using dark-field imaging analysis [86] (Figure 2b). The conventional dark-field imaging and its spectrographic analysis have some disadvantages such as measurement of only few nanoparticles at once, time-consuming process, and hard to focus on the nanoparticles inside the cell because they are easily floating. Thus, an algorithm (bias-modified fuzzy C-means; BM-FCM) was applied to analyze the LSPR-based dark-field image of the Au nanoparticles inside the cells. The BM-FCM made it possible to eliminate the disturbance of scattering light and segmentation of the images as the pixels. As seen in Figure 2b, the distribution of Au nanoparticles in the processed image was clearly verified compared to the conventional method. In addition, they exhibited that this analytical method allowed to get detailed information for monitoring the distribution of NADH in cancer cells for estimating efficiency of cancer drugs. Based on these studies, MNP-mediated nanoparticles could provide the in situ intercellular analytical platforms in a highly sensitive and selective manner.

### 3.2. LSPR-Based Analytical Platform with Metallic Nanostructures on Cell Chip for Extracellular Signal Detection

Recently, there have been a lot of cell chip platforms to allow in situ analysis of the several biomaterials such as DNA, miRNA, mRNA, cytokines, etc., in live cells due to their versatility for application to toxicological tests, drug screening, and investigation of stem cell fate [89,90,91,92,93,94]. Diverse detection methods have been integrated into the cell chip; however, most of the analytical methods require labeling of the signaling molecules such as fluorophore for the immunofluorescence or other fluorescence-based methods. In addition, the need for labeling for the biomolecular analysis will affect the cell metabolism even if the labeling does not have cytotoxicity for the culture periods. On the other hand, LSPR-based detection platforms could measure the biomolecules without the labeling steps. LSPR can provide a real-time monitoring system against external stimuli easily. Moreover, as previously mentioned, LSPR offers unique dark-field imaging of the cells simultaneously [86,95]. Zhu et al. developed LSPR-based biosensor-integrated adipose-tissue-on-chip for multiplexed cytokines secretion analysis [87] (Figure 2c). Adipocyte was cultured on the center of the chip with the macrophages, and the four surrounded circle rings functionalized by the Au nanorod and anti-cytokines, which capture each cytokine (IL-4, IL-6, IL-10, and TNF-α, respectively). When cytokines are secreted from the adipocytes, cytokine-containing medium flowed from the center to the suburb of the chip, and the antibodies captured the targeted cytokines. Since the dielectric constant of the Au nanorods was changed once the cytokine was captured, the LSPR signals were altered and it could be translated to dark-field imaging. Using this platform, monitoring of the adipose tissue was possible, such as initiation, differentiation, and maturation. In addition, it enabled simultaneous multiplexed measurements of pro-inflammatory and anti-inflammatory cytokines, closely related to the obesity progression. Other groups exhibited the 3D multilayered Au nanosquares array using reversal nanoimprint lithography [96]. A 3D structure was superior to the EM field by plasmonic effect on the Au nanostructure compared to a 2D structure. This 3D multilayered Au nanostructure showed high sensitivity (442 nm/RIU), could detect low concentration of A549 cells, whereas Au nanoparticle-based biosensor exhibited sensitivity of 120 nm/RIU with microfluidic device. Using this platform, A549 cells were analyzed by the resonance peak shifts at 581 and 805 nm. Peak shifts were proportional to the cell concentration of A549 and could detect 5 × 10^3^/mL cells. In addition, it could detect the DNA hybridization at low concentration 10^−14^ M of the complementary target DNA, and the largest peak shift (82 nm) was observed at 10^−7^ M. Luo et al. developed the U-shaped fiber optic biosensor for the detection of cancer cells and its specific surface protein, *N*-glycan [97]. To place the LSPR property on the U-shaped fiber optic, Au nanoparticles were functionalized on the surface by the chemical linker. In addition, Con A was immobilized to the capture of the cancer cells and *N*-glycan. Fiber optic was easily used as a light source for induction of LSPR and a detector to measure the absorption value. Through this system, cancer cells were measured with a 30 cells/mL limit of detection (LOD), which was superior to the non-Au functionalized one, showing approximately 29-times-lower LOD and good linearity in a wide range of 1 × 10^2^–1 × 10^6^ cells/mL of MDA-MB-231 cancer cells with a label-free and in situ manner. Usukura et al. presented the high axial-resolution fluorescence imaging using the Ag nanoparticle-mediated LSPR effect [88] (Figure 2d). For effective and high-resolution cell imaging, a two-dimensional Ag nanosheet, composed of Ag nanoparticles, was utilized. In this study, the LSPR effect showed tremendously high axial confinement and virtually high lateral resolution for the high-speed cell imaging. The Ag nanosheet could improve the fluorescence signals and offer high signal-to-noise ratio images at the cell–nano interface. As a result, fluorescent dye-labeled cells could be more clearly observed than the conventional method, such as total internal reflection fluorescence (TIRF) microscopy. In summary, metal nanomaterials have been used to enhance the signals from the biological event at the intracellular level through the LSPR effect with chip-based platforms.

**Table 2:** LSPR-based analytical platforms for the cell analysis with the metallic nanomaterials.

Metallic Nanomaterials		Mechanism	Target	Function	Ref.
**Solution-based metallic nanoparticles**	Crown nanoparticle plasmon rulers (Au nanoparticle)	Scattering spectra by aggregation–dissociation	Caspase-3	Continuously monitoring of caspase-3 activity in live cells for over 2 h	[84]
	Plasmonic nanohalo (Large and small Au nanoparticle)	Scattering spectra by aggregation–dissociation	Survivin mRNA	Detecting and imaging survivin mRNA by dark-field analysis in living cells	[85]
	Au nanoparticle	Scattering and absorption spectra of Au nanoparticle, bias-modified fuzzy C-means algorithm	HeLa cells, NADH	Fast and high-throughput analysis of cell imaging and presence and location of important biological molecules	[86]
**Nano-platform onto the cell chip**	Circular sensing array (Au nanorod)	Resonance peak shift by LSPR	IL-6, TNF-α, IL-10, IL-4	Multiplexed measurements of cytokines from adipocyte and macrophage	[87]
	3D multilayered Au nanosquare	Resonance peak shift by LSPR	A549 cells, DNA	5 × 10^3^ cells mL^−1^ in 2 μL and c 10^−14^–10^−7^ M DNA could be detected	[96]
	U-shaped fiber optic with Au nanoparticles	Increase of absorption value	*N*-Glycan	Label-free and in situ cytosensing of *N*-glycan expression on the cell surface	[97]
	Silver nanoparticle sheet	Confinement and enhancement of the fluorescence by LSPR	RBL-2H3 cell, NIH-3T3 cell	Obtaining high-quality nanointerfacial cell images	[88]

## 4. SERS-Based Analytical Platform on Cell Chip Using MNPs

Raman spectroscopy is a noninvasive method through the detection of specific chemical structures and biomolecules by the individual scattering phenomena with incident light [98,99]. Raman spectra can be shifted due to the scattered photons from the chemistry of each molecule. Therefore, specific Raman shifts from the biomolecules can be characterized as spectra for the determination of the target analytes. By way of this analytical method, biomarkers have been measured in a precise manner. However, typical Raman intensity from the biomolecules showed very low intensity for the measurement of the low-level concentration of the biomolecules, which necessitates highly sensitive detection at the intra/extracellular level for pharmaceutical applications. On the other hand, SERS is the signal-enhancing technique affecting surface plasmon effects on the surface of MNPs to determine the low concentration of biomolecules [100,101,102]. To induce the SERS effect, several MNPs have been used with various shapes such as circle or triangle, where the EM fields related to the plasmon modes are hardened to increase the Raman vibrational signals from the biomolecules [103,104]. With enhancement factors of the order of 10^14^ possible, this makes the SERS analytical method an extraordinarily sensitive detection. In this section, recent studies related to SERS-based intra/extracellular analytical platforms are briefly introduced (Table 3).

### 4.1. Metallic Nanoparticle-Based SERS Sensing Strategies for Intracellular Signal Detection

**Figure 3:**
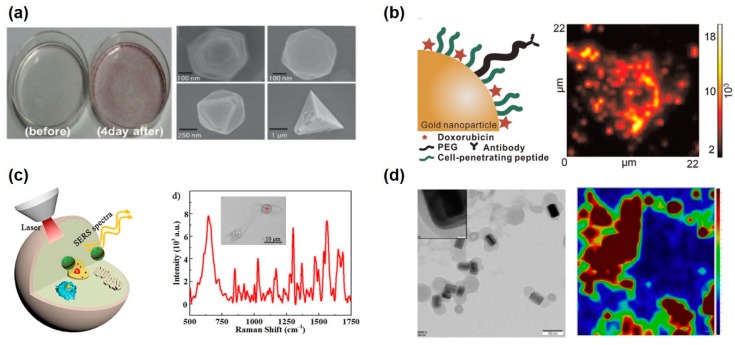
SERS-based intracellular analysis using metallic nanoparticles. (**a**) Synthesis of Au nanoparticle in the HeLa cells after incubation with a gold chloride solution and they were measured by the SERS analytical method. Reproduced with permission [107]. Copyright 2013, WILEY-VCH. (**b**) Biohybrid Au nanoparticles and time-dependent monitoring of the nanoparticle’s specific targeting, cellular uptake, release of doxorubicin in the cancer cells by glutathione. Reproduced with permission [108]. Copyright 2015, Elsevier. (**c**) Ag-graphene oxide nanoparticles for the excellent SERS sensing capability biocompatible nanoprobes for intracellular biosensing of the releasing doxorubicin. Reproduced with permission [109]. Copyright 2018, ACS Publications. (**d**) 4-aminothiophenol (ATP) modified-Au–Ag core-shell nanorods for the improvement of SERS effect and SERS tracking inside live cancer cells. Scale bar is 100 nm. Reproduced with permission [111]. Copyright 2019, Elsevier.

MNPs can assist with the detection of intracellular changes through the detection of specific biomolecules or ionic changes for the pH sensing by the SERS-based analysis. Zong et al. developed the intracellular pH sensor using p-aminothiophenol (pATP), a Raman reporter, functionalized hydrochloric acid (HCl)-treated Au nanorods [105]. A HCl-treated Au nanorod has the advantages of not only reduced cytotoxicity but also direct contact between the reporters and the intracellular surroundings, which could detect pH value by the interaction between pATP and Au nanorod. In the alkaline condition, pATP-functionalized Au nanorods bind to each other through the form of pATP, 4, 40-dimercaptoazobenzene (DMAB). Their conformational changes induced the alteration of the ratio of 1432 to 1076 cm^−1^. The intracellular pH value of HeLa cells was successfully measured, from 3 to 8 pH. Another study showed the determination of neuronal differentiation using nuclear localization signal peptides (NLS)-functionalized Au nanoparticles in a human neuroblastoma cell line using SERS analysis [106]. The neural progenitor cells have different biological properties such as the DNA/RNA ratio and protein expression levels, comparing differentiated cells. The authors succeeded to distinguish between the Raman spectrum of differentiated cells and progenitor cells using the principal component analysis for whole cell scans. Though there were some analytical processes for the principal component analysis, it could segregate undifferentiated from differentiated cells with a simple endocytosis method of Au nanoparticles. MNP-based SERS signals have also been measured in living cells. One study exhibited the formation of the MNPs inside the living cells with diverse shapes such as circle, triangle, and square, and they were detected by the SERS analytical method [107] (Figure 3a). The result demonstrated that treatment of different cell lines with metal ions resulted in forming the different shapes of MNP due to the reduction of metal ions in the living cells. In addition, formed Au nanoparticles in the living cells exhibited enhanced Raman spectra with a large-wavelength laser, which could minimize fluorescence interference and photo-toxicity. Another study showed in situ monitoring of releasing drug from the nanoparticles inside the cells using SERS-based analysis [108] (Figure 3b). Au nanoparticles were utilized as the intracellular delivery cargo with doxorubicin, cell-penetrating peptide, and anti-HER2 antibody. After the endocytosis of Au nanoparticle to the HER2-positive cancer cells (SK-BR-3), doxorubicin was released due to the intracellular glutathione. According to the SERS results, 90.23% of the doxorubicin was released within 24 h of particle treatment. Zeng et al. developed graphene oxide-coated Au nanoparticles without adding any surfactant, which caused low efficiency of the SERS signal, and it applied to the intracellular drug delivery with doxorubicin [109] (Figure 3c). The graphene oxide-coated Au nanoparticle showed excellent SERS signal because graphene oxide also enhanced Raman signal by chemical enhancement and was used as biocompatible nanoprobes for intracellular biosensing and doxorubicin delivery with π–π interaction between graphene oxide and doxorubicin. For the enhancement of SERS intensity, gold and Au were used as a core-shell nanoparticle formation labeled with Raman probe, para-mercaptobenzoic acid (4MBA) molecules [110]. This bimetallic core-shell nanostructure showed the presence of two interacting LSPR modes and high SERS activity. For the no anti-proliferative effects, chitosan was coated on the core-shell nanoparticles and it could be used to measure the intracellular pH through the measurement of SERS signal, from pH 4.3 to 7.5. Ziang et al. applied zeolitic imidazolate framework (ZIF-8) as a coating agent to the gold-Au core-shell nanoparticles for the enhancement of SERS intensity [111] (Figure 3d). This nanoparticle could also be loaded with doxorubicin and targeting moiety (folic acid) for cancer treatment and intracellular imaging. The resulting nanoparticles possess a strong SERS effect, high drug loading capacity, and excellent biocompatibility. In addition, it was used as optical and Raman imaging simultaneously and cancer treatment by doxorubicin. Otherwise, several MNPs could enhance the SERS signal from the intracellular region for the effective analysis of biological alteration.

### 4.2. SERS-Based Analytical Platform with Metallic Nanostructures on Cell Chip for Extracellular Signal Detection

**Figure 4:**
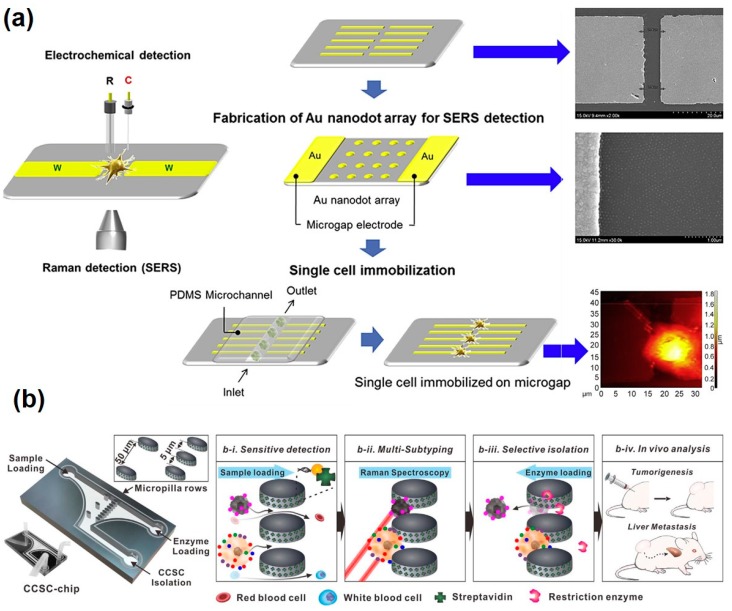
SERS-based extracellular analysis using metallic nanomaterials. (**a**) Single cell-based chip to investigate redox state of PC12 cells using SERS and linear sweep voltammetry (LSV) techniques with Au nanodot array and microgap structure. Scale bar of the right-upper image is 20 µm and the right-lower image is 1 µm, respectively. Reproduced with permission [115]. Copyright 2015, Elsevier. (**b**) Raman-active nanoprobe-based chip platform with the capability of simultaneous detection, isolation, and further analysis of circulating cancer stem cells (CCSCs) and various circulating tumor cells (CTCs) subtypes though Raman imaging. Reproduced with permission [116]. Copyright 2018, ACS Publications.

In addition to the intracellular analysis using the SERS-based analytical method, there have been extracellular analytical platforms integrated with metal nanostructures on analytical chips to allow in situ monitoring of the live cells. Neural stem cell differentiation was determined using three-dimensional graphene oxide-encapsulated gold nanoparticles by SERS signal measurement [112]. The graphene oxide-encapsulated gold nanoparticles were deposited on the chip surface, and the neural stem cells were maintained and differentiated. Undifferentiated cells had high numbers of C=C bonds; on the other hand, differentiated neuronal cells exhibited low numbers of C=C bonds due to the saturated cell membranes. Using this phenomenon, Raman shift can be measured with different intensities of the specific peaks (around 1656 cm^−1^) between undifferentiated and differentiated neuronal cells. In addition, electrochemical analysis was conducted simultaneously using the redox property of the neural stem cells. In result, graphene oxide-encapsulated gold nanoparticles combined with SERS and electrochemical techniques were successfully monitored through stem cell differentiation. Moreover, an Au nanodot array with Au microgap electrode was developed for single cell analysis using SERS and linear sweep voltammetry (LSV) analysis simultaneously [113] (Figure 4a). This combined platform could measure the intracellular and extracellular redox state of PC12 cells, which is dopaminergic cell, available for both bulk and single PC12 cell. It is expected that this spectroelectrochemical cell chip platform can be a very powerful in situ tool for monitoring intracellular and extracellular changes with multi and single cell level regarding their redox environment. The breast cancer cell using Ag nanostar modified ITO substrates were also characterized. [114]. Compared with an Au nanopattern modified ITO substrate, Raman signals from the Ag nanostar modified ITO substrate showed significantly increased signals. Using this Ag nanostar platform, SK-BR-3 and MCF-7 breast cancer cells were determined to different Raman peaks. The dominant peaks of the MCF-7 were at 1030, 1060, and 852 cm^−1^, and SK-BR-3 showed significant peaks at 1377 and 1490 cm^−1^. Au nanopatterned ITO substrate was also utilized for the distinguishing of breast cancer subtype by using SERS analysis [115]. Two different breast cancer cell lines (MDA-MB-231 and MCF-7) were determined by the measurement of chemical components such as nucleic acids, fatty acids, chemical bonds, etc. Raman spectrum by SERS analysis of the MDA-MB-231 and MCF-7 exhibited different shapes and peaks so the classification of the cells could be relatively easy with the nondestructive method by measurement of Raman spectrum. Noninvasive analysis of the circulating cancer stem cells (CCSCs) was successfully conducted with microfluidic chips and Raman reporter-labelled Au nanoparticles [116] (Figure 4b). Solution-based analyte with several cells was flown into the cell and micropillars separated circulating cells and other small biomolecules. Raman reporter-labelled Au nanoparticles were selectively bound to the CCSC surface and it could confirm the subtyping of specific cells, which was characterized by the surface marker such as HER2, CD133, EGFR, EpCAM, and MUC-1. In addition, separated cells could be isolated by the loading of restrict enzyme with opposite flow for the detachment of CCSC on the micropillar. CCSCs were detected (90% efficiency), and selectively isolated CCSCs (93% accuracy) were employed for both in vitro and in vivo tumor phenotyping to identify the tumorigenicity of the CCSCs.

## 5. Future Perspective and Conclusions

To date, human-relevant models of several diseases have been studied to understand the disease mechanism and validate the therapeutic effect of the drug candidates. Even though animal models have been utilized as a systemic in vivo surrogate estimating human disease pathophysiology and drug testing platform, inter-species differences regarding drug responses or metabolism between animals and humans causes serious problems [117]. Alternatively, in vitro cell-chip-based models have been developed, but could not emulate the organ-level physiological properties. Therefore, the development of a revolutionized new model of humans is a critical unmet need. The concept of the organ-on-a-chip has been raised for development of an in vitro cell-based model that is more relevant to the actual human physiology at an organ-level by using cell-chip technology with microfluidics [118,119,120]. Generally, organ-on-a-chip consists of two or more differentiated cells and microfluidic channels with fluidic flow, which mimic the shear stress and supply the nutrients for maintaining long periods. Through these microfluidic-based systems, various human organs have been reconstructed, and they have verified the similarity to each organ function, such as lung, liver, heart, intestine, brain, etc. [118,121,122,123,124,125]. By taking advantage of the organ-on-a-chip, the ultimate goal of the cell-based system is mimicking specific diseases with patient-derived cells and utilizing it as a drug screening tool for personalized medicine. To realize this goal, in situ monitoring methods are mandatorily required to be integrated into the mimicking organ-on-a-chip device. The current cellular condition or signal monitoring methods on cell-chip platforms are profoundly weighted on immunofluorescence. However, immunofluorescence requires a fixation process, which means that in situ monitoring of cells is not possible. Moreover, immunofluorescence can only measure the specific proteins with low sensitive and time-consuming manner. In the case of some blood-brain-barrier-integrated organ-on-a-chip devices, some biosensing function was integrated. The transepithelial electrical resistance (TEER) value was monitored to observe the change against external stimuli such as cytotoxic materials with micro-sized electrodes [126]. However, observation of the TEER value provides only limited information, such as tight junction protein and cell viability. As previously mentioned, metallic nanomaterials could be applied to measure biomolecules in live cells, enabling in situ monitoring in a highly sensitive and selective manner. In addition, it can offer chances to select diverse analytical methods such as fluorescence, SERS, and LSPR-based detections, which makes it feasible to integrate sensing modules into organ-on-a-chip platforms. Metallic nanomaterials can also offer the multi-analytes detection that is necessary to the organ-level analysis. Therefore, we expect that metallic nanomaterial-based biosensing systems have significant potential to analyze the organ-level changes into the organ-on-a-chip for the pathophysiology and discovery of precise and effective drugs.

In summary, MNPs can be integrated with cell chip platforms to improve sensing abilities such as high sensitivity, high selectivity, rapid and straightforward detection, in situ monitoring, multi-detection, etc. Here, we report the current approaches of in vitro MNP integrated cell chip platforms with several analytical methods, which are fluorescence, SERS, and LSPR-based measurement. Each of the methods has their special properties, and they are easily utilized to improve the sensing performances. MNPs could facilitate advanced sensing systems due to their significant EM property compared to other nano-sized materials. In the near future, it is expected that advanced platforms in organ-on-a-chip or more emulating systems for reconstructing human microenvironmental systems with metallic-nanomaterial-based sensing module will provide a robust in vitro drug development platform that can replace in vivo animal models and exploit in vitro personalized analysis in the pharmaceutical applications.

## Figures and Tables

**Table 3:** Surface-enhanced Raman spectroscopy (SERS)-based analytical platforms for the cell analysis with the metallic nanomaterials.

Metallic Nanomaterials		Mechanism	Target	Function	Ref.
**Solution-based metallic nanoparticles**	Au nanorod	Change of SERS spectra by aggregation–dissociation	pH	Measurement of intracellular pH from pH 3 to 8	[105]
	Au nanoparticle	Scattering spectra by aggregation–dissociation	Cell nucleus	Segregation of undifferentiated from differentiated cells in a human neuronal cell line using PCA analysis	[106]
	Au and Ag nanoparticle	SERS spectrum from different shape and size of intracellular-synthesized Au nanoparticles	HEK293T cells	Green synthesis method for production of different metal nanoparticles inside living cells and detection by SERS	[107]
	Au nanoparticle	Monitoring of SERS intensity of doxorubicin inside the cancer cell	Doxorubicin	Label-free in situ monitoring of intracellular anti-cancer drug-releasing by Au nanoparticles based on SERS	[108]
	Graphene oxide coated silver nanoparticle	Monitoring of SERS intensity of doxorubicin inside the cancer cell	Doxorubicin	SERS effect for monitoring of the loading and releasing of doxorubicin attached to the surface of nanoparticle	[109]
	Au-Ag core-shell nanoparticle	Shift of the Raman peaks of 4MBA by the intracellular pH value	pH	High SERS activity of the bimetallic nano-construct with 4MBA for the intracellular pH measurement	[110]
	Au-Ag nanorod-ZIF-8 core-shell nanoparticles	SERS effect of the 4-ATP on the surface of nanoparticle	Cancer cells	Targeted SERS imaging of the cancer cells	[111]
**Nano-platform onto the cell chip**	3D graphene oxide-encapsulated Au nanoparticle	Change the SERS intensity by the C=C bond of differentiated and undifferentiated cells	Number of C=C bonds	Detection of the differentiation potential of neural stem cells based on SERS	[112]
	Au nanodot array and microgap electrode	Change of the SERS spectrum by oxidization and reduction of cell	PC12 cell	Measurement of intracellular and extracellular redox state of PC12 cells using SERS and electrochemical techniques	[113]
	Ag nanostar patterned ITO substrate	Measurement of biomolecules inside the cells by SERS	SK-BR-3, MCF-7	Biomolecular detection and characterization of different breast cancer cell lines	[114]
	Au nanosphere deposited ITO substrate	Measurement of biomolecules inside the cells by SERS	MDA-MB-231, MCF-7	Biomolecular detection and characterization of different breast cancer cell lines	[115]
	Au nanoparticle	Detection of Raman reporter-labelled Au nanoparticle on the different kind of CCSC	MCF-7, MDA-MB-231, SK-BR-3, Humanbreast CCSC	Nanoparticle-mediated Raman imaging for CCSC characterization which profiles based on the surface marker expression phenotype	[116]
